# HIV-1 diversity in an antiretroviral treatment naïve cohort from Bushbuckridge, Mpumalanga Province, South Africa

**DOI:** 10.1186/s12985-015-0244-1

**Published:** 2015-02-13

**Authors:** Patrick Wela Msimanga, Efthyia Vardas, Susan Engelbrecht

**Affiliations:** Division of Medical Virology, Department of Pathology, Faculty of Medicine and Health Sciences, Stellenbosch University, Tygerberg Hospital, P.O. Box 241, Cape Town, 8000 South Africa; Lancet Laboratories, P.O. Box 8475, Johannesburg, 2000 South Africa; National Health Laboratory Services (NHLS), Western Cape Region, Tygerberg Hospital (Coastal), Tygerberg, Cape Town South Africa; Current Address: National Department of Health, Civitas Building, Corner Thabo Sehume and Struben Streets, P.O. Box X828, Pretoria, 0001 South Africa

**Keywords:** HIV-1 diversity, Phylogenetic analysis, Transmitted resistance, Mpumalanga Province

## Abstract

**Background:**

South Africa has a generalized and explosive HIV/AIDS epidemic with the largest number of people infected with HIV-1 in the world. Molecular investigations of HIV-1 diversity can help enhance interventions to contain and combat the HIV/AIDS epidemic. However, many studies of HIV-1 diversity in South Africa tend to be limited to the major metropolitan centers and their surrounding provinces. Hardly any studies of HIV diversity have been undertaken in Mpumalanga Province, and this study sought to investigate the HIV-1 diversity in this province, as well as establish the occurrence and extent of transmitted antiretroviral drug resistance mutations.

**Methods:**

HIV-1 *gag* p24, *pol* p10 and p66/p51, *pol* p31 and *env* gp41 gene fragments from 43 participants were amplified and sequenced. Quality control on the sequences was carried out using the LANL QC online tool. HIV-1 subtype was preliminary assigned using the REGA 3.0 and jpHMM online tools. Subtype for the *pol* gene fragment was further designated using the SCUEAL online tool. Phylogenetic analysis was inferred using the Maximum Likelihood methods in MEGA version 6. HIV-1 antiretroviral drug resistance mutations were determined using the Stanford database.

**Results:**

Phylogenetic analysis using Maximum Likelihood methods indicated that all sequences in the study clustered with HIV-1 subtype C. The exception was one putative subtype BC unique recombinant form. Antiretroviral drug resistance mutations K103N and E138A were also detected, indicating possible transmission of anti-retroviral drug resistance mutations.

**Conclusions:**

The phylogenetic analysis of the HIV sequences revealed that, by 2009, patients in the Bushbuckridge, Mpumalanga were predominantly infected with HIV-1 subtype C. However, the generalized, explosive nature of the HIV/AIDS epidemic in South Africa, in the context of extensive mobility by South Africans who inhabit rural areas, renders the continued molecular monitoring and surveillance of the epidemic imperative.

**Electronic supplementary material:**

The online version of this article (doi:10.1186/s12985-015-0244-1) contains supplementary material, which is available to authorized users.

## Background

Human immunodeficiency virus (HIV), the etiological agent of acquired immunodeficiency syndrome (AIDS), was first isolated more than 30 years ago [1]. By 2013, an estimated 35 million people were living with HIV-1 globally, of which 24.7 million were living in sub Saharan Africa [2]. During this time period, the HIV-1 prevalence in South Africa was 12.2% (6.4 million people), with 469 000 new infections occurring, suggesting that the epidemic is not only generalized, but also explosive [3].

The HIV-1 epidemic in South Africa is characterized by limited subtype diversity with subtype C accounting for the majority of infections [4,5]. Other non-C subtypes, particularly subtypes B and D, have also been identified [6-8] as well as the occasional unique recombinant forms (URFs) [9-15]. Molecular epidemiological investigations in South Africa have largely focused on provinces with major metropolitan centers such as Johannesburg in Gauteng, Cape Town in the Western Cape and Durban in Kwa-Zulu Natal. No subtype information is available for the Eastern Cape, North West and Northern Cape provinces and limited information is available for the Free State, Limpopo and Mpumalanga Provinces. HIV-1 prevalence in South Africa is also characterized by extreme heterogeneity and there is considerable variation in prevalence amongst the different provinces and districts in each province [16]. The highest prevalence is in Kwa-Zulu Natal with the lowest in the Western Cape Province. South Africa not only has a generalized and explosive HIV/AIDS epidemic, its impact also varies significantly in terms of race, age, gender, and between regions of the country, with poor, young, African women in rural Kwa-Zulu Natal bearing a disproportionate burden of HIV infection [16].

The overall HIV prevalence in Mpumalanga in 2012 was 35.6% [16]. The province consists of 3 districts: Ehlanzeni, Nkangala and Gert Sibande. The Bushbuckridge Local Municipality in the Ehlanzeni District in Mpumalanga Province is a predominantly rural, impoverished area, with only 14% of the adult population employed and over 85% of households living below the house hold subsistence level. Half of males and 14% of females between the ages of 25 and 59 are long-term migrant workers and provide a source of remittances, which comprise the largest proportion of the income of the population of Bushbuckridge [17].

Molecular investigations of HIV diversity can help enhance interventions to contain and combat the HIV-1 epidemic. With this study, we investigated for the first time, HIV-1 diversity in Bushbuckridge, Mpumalanga, as well as the possible occurrence and extent of transmitted antiretroviral drug resistance mutations.

## Methods

### Study population and RNA extraction

**Figure 1:**
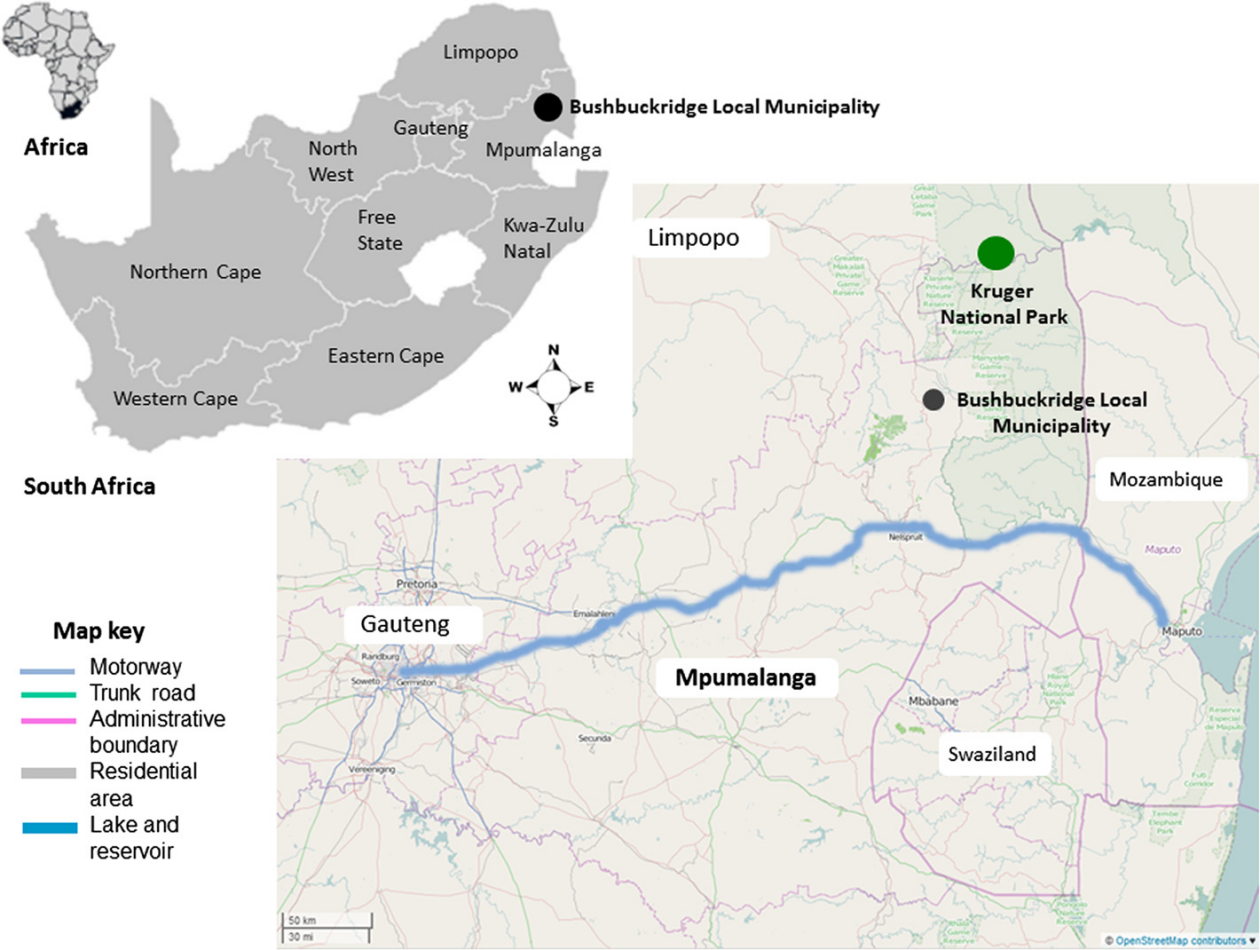
**Geographical location of samples collected in this study.** The South African map with 9 provinces is indicated and the Bushbuckridge local municipality in the Ehlanzeni district of Mupumalanga is enlarged. The “Maputo corridor” or N4 trunk roadway is highlighted in blue.

In preparation for HIV prevention trials, a cohort was developed for enrollment. Ethics approval were obtained from the Human Research Ethics Committees (HRECs) from the University of the Witwatersrand (M061129) and Stellenbosch University (N11/02/054), following internationally recognized guidelines. The entry point for this cohort was via a free voluntary counseling and testing service. After HIV testing, individuals were offered the opportunity to be part of the pre-screening cohort. Both HIV negative and HIV positive individuals were allowed to join the cohort in preparation for preventative and therapeutic HIV vaccine trials. Fifty-one samples were obtained with informed consent as part of this pre-screening protocol from 43 HIV positive participants in Bushbuckridge, Mpumalanga (Figure 1). RNA was extracted from stored plasma samples using a QIAamp MinElute Virus Spin Kit in a QIAcube automated extractor (QIAGEN, Dusseldorf, Germany), according to the manufacturer’s instructions. RNA samples were stored at −70°C until used.

### Reverse transcriptase polymerase chain reaction (RT-PCR) of HIV-1 gene fragments

Four genomic regions were targeted for amplification: the *gag* p24 region (HXB2 nucleotides 1248 to 1707); a part of the *pol* gene, that includes the Protease (PR) and a partial segment of the Reverse Transcriptase (RT) region (HXB2 nucleotides 2114 to 3335), the *Integrase* (IN) region (HXB2 nucleotides 4202 to 5096) and the partial *env* gp 41 region (HXB2 nucleotides 7877 to 8282). PCR amplification and purification was done using previously described primers and methods for the partial *gag, pol integrase* (IN) and *env* [18] genes. The partial *pol* PR/RT gene was also amplified using primers and a method previously described [19,20]. Briefly, cDNA synthesis and first round PCR amplification was done with the Access-RT PCR system (Promega, Wisconsin, USA), while second round nested PCR amplification was done with the GoTaq DNA polymerase system (Promega, Wisconsin, USA). The oligonucleotide primers used in the amplification of the gene fragments are listed in Table 1.

**Table 1:** **Primers used in the amplification of the partial HIV-1 gene products**

**Primers** ^**#**^	**Primer sequence 5’ to 3’**	HXB2 position*
p24-1 (*gag*)	AGYCAAAATTAYCCYATAGT	1174 - 1193
p24-2 (*gag*)	AGRACYTTRAAYGCATGGGT	1237 - 1256
p24-6 (*gag*)	TGTGWAGCTTGYTCRGCTC	1673 - 1654
p24-7 (*gag*)	CCCTGRCATGCTGTCATCA	1844 - 1826
PR-5′ prot-1 (*pol*)	TAATTTTTTAGGGAAGATCTGGCCTTCC	2082 - 2109
PR-5′ prot-2 (*pol*)	TCAGAGCAGACCAGAGCCAACAGCCCCA	2136 - 2163
RT-NE135 (*pol*)	CCTACTAACTTCTGTATGTCATTGACAGTCCAGCT	3334 - 3300
RT-MJ4 (*pol*)	CTGTTAGTGCTTTGGTTCCTCT	3420 - 3399
Poli 5 (*integrase*)	CACACAAAGGRATTGGAGGAAATG	4162 - 4185
poli7 *(integrase*)	AACAAGTAGATAAATTAGTCAGT	4186 - 4208
poli6 (*integrase*)	ATACATATGRTGTTTTACTAARCT	5130 - 5107
poli8 (*integrase*)	TAGTGGGATGTGTACTTCTGAAC	5217 - 5195
JH41 (*env*)	CAGCAGGWAGCACKATGGG	7798 - 7816
Env 27 F (*env*)	CTGGYATAGTGCARCARCA	7861 - 7879
Menv19 R (*env*)	AARCCTCCTACTATCATTATRA	8299 - 8278
JH38 (*env*)	GGTGARTATCCCTKCCTAAC	8346 - 8365

^#^Primer References [18,20].

*Nucleotide position of the primer according to the HXB2 sequence (K03455) numbering.

### Sequencing of HIV-1 gene fragments

The cycle sequencing reactions of the partial gene fragments were done with the Big Dye® Terminator v 3.1 Cycle Sequencing Kit (Applied BioSystems, Foster City, CA, USA) and run on an ABI Prism 3130xl Genetic Analyzer (Applied Biosystems, Foster City, CA, USA), according to the manufacturer’s instructions. Both strands were sequenced using overlapping primers. Sequencher v 5.1 (Gene Codes Corporation, Ann Arbor, MI, USA) was used to assemble the trace data into contiguous fragments, which were then verified, edited and saved as text files for subsequent analysis. All sequences were checked for quality assurance using the Los Alamos HIV-1 Sequence Quality Analysis tool (http://www.hiv.lanl.gov/content/sequence/QC/index.html) before further analyses and submission to GenBank.

### Preliminary HIV-1 subtyping using online tools

The REGA HIV-1 subtyping Tool Version 3.0 (http://dbpartners.stanford.edu:8080/RegaSubtyping/stanford-hiv/typingtool) was used to preliminary subtype the sequences [21]. To detect recombinants, we used the jumping profile Hidden Markov Model (jpHMM-HIV) tool (http://jphmm.gobics.de) [22]. Subtype Classification Using Evolutionary Algorithms (SCUEAL) was used to test for both intra and inter subtype recombinants in the partial HIV-1 *pol* and IN sequences (http://www.datamonkey.org/dataupload_scueal.php) [23].

### Phylogenetic inference using Maximum likelihood (ML)

The 2010 HIV-1 Group M reference sequence dataset (n = 39), from the LANL database (http://www.hiv.lanl.gov/), was used to subtype our sequences, using phylogenetic inference. Multiple sequence alignments, comprising the partial *gag*, *pol* and *env* sequences, and the reference dataset, were constructed using MAFFT v7.017 [24] as implemented in Geneious version R7 created by Biomatters (http://www.geneious.com). These multiple sequence alignments were subsequently codon aligned using Codon Alignment v1.1.0 (http://www.hiv.lanl.gov/content/sequence/CodonAlign/codonalign.html) and manually checked.

The phylogenetic trees for the different HIV-1 genetic fragments were inferred using ML methods implemented in MEGA version 6 [25]. To find the most appropriate evolutionary model for phylogenetic inference, we used Model Selection (ML) as implemented in MEGA [25]. For each model, BIC scores (Bayesian Information Criterion), AICc value (Akaike Information Criterion, corrected), Maximum Likelihood value (lnL), and a number of different parameters were presented. Models with the lowest BIC scores were considered to describe the substitution pattern the best [25]. For the partial *pol* PR/RT region, the *Integrase* (IN) region and the partial *env* gp 41 region, the BIC, AICc and lnL scores indicated that the General Time Reversible model of evolution with Gamma distribution and invariant rate among sites (GTR + G + I), was the best model. For the *gag* region, the lnL method indicated the use of the GTR + G + I model and BIC and AICc indicated the use of the TN93 + G + I model. All nucleotide positions in the alignments with less than 95% site coverage were eliminated, thus fewer than 5% alignment gaps, missing data, and ambiguous bases were allowed at any position. The reliability of the inferred trees was evaluated using bootstrap resampling and branches with a bootstrap value of 70% or greater were considered reliable (n = 100) [26].

### HIV-1 antiretroviral drug resistance mutations using HIVdb

HIV-1 PR and RT antiretroviral drug resistance mutations were determined using the Stanford University HIV Drug Resistance Database (HIVdb), http://www.hivdb.stanford.edu/ [27].

### GenBank accession numbers

GenBank accession numbers of the *gag* sequences were KM218392 to KM218428; *pol* sequences, KM218448 to KM218460; *integrase* sequences, KM218429 to KM218447 and for the *env* sequences, KM218357 to KM218391.

## Results

### Demographic information

The demographic and clinical information of the cohort, together with the subtyping, are summarized in Table 2. The study involved 51 plasma samples, collected from 43 participants in Bushbuckridge, between February and July 2009. Forty samples were collected at the recruitment visit and 11 samples at visit one. Only one sample per participant was included in the study. All participants, except for 0064A and 0206A, were female and none were on HIV-1 antiretroviral treatment. The average age of the cohort was 26.7 years and ranged from 16 to 41 years. The CD4 lymphocyte count ranged from 105 to 1263 with an average of 450.

**Table 2:** **Demographic and clinical information of the participants**

**Study number**	**Collection Date**	**Age**	**Gender**	**CD4 count**
0005A	23/04/2009	16	Female	466
0022A	24/04/2009	32	Female	731
0038	16/03/2009	31	Female	680
0039	17/03/2009	19	Female	307
0040	23/03/2009	23	Female	506
0042A	19/02/2009	26	Female	511
0064A	16/03/2009	25	Male	105
0066A	17/03/2009	22	Female	583
0073	02/04/2009	24	Female	366
0081A	08/04/2009	23	Female	437
0085	14/04/209	32	Female	154
0092A	16/04/2009	23	Female	137
0097A	17/04/2009	25	Female	243
0098A	20/04/2009	24	Female	261
0101A	20/04/2009	30	Female	790
0103	20/04/2009	22	Female	191
0116A	29/07/2009	26	Female	150
0119A	29/04/2009	28	Female	403
0122A	30/04/2009	33	Female	262
0123A	04/05/2009	34	Female	335
0130A	06/05/2009	29	Female	387
0132A	07/05/2009	25	Female	489
0134A	11/05/2009	26	Female	792
0135A	11/05/2009	23	Female	1263
0136A	11/05/2009	23	Female	1192
0143A	25/05/2009	30	Female	353
0147A	27/05/2009	19	Female	785
0152A	03/06/2009	20	Female	691
0165A	22/06/2009	32	Female	198
0173A	29/06/2009	27	Female	367
0185A	02/07/2009	30	Female	311
0189	20/07/2009	33	Female	560
0190A	06/07/2009	37	Female	229
0192A	06/07/2009	41	Female	522
0193A	06/07/2009	29	Female	313
0198A	07/07/2009	27	Female	217
0199A	07/07/2009	30	Female	219
0203A	08/07/2009	25	Female	401
0204A	08/07/2009	21	Female	349
0206A	08/07/2009	31	Male	314
0207A	08/07/2009	22	Female	733
0211	08/07/2009	31	Female	312
0215A	09/07/2009	22	Female	726

### PCR amplification, sequence data and quality assurance

PCR amplification was successful for most of the samples, with 93% (n = 40) of the partial *gag* p24 gene, 48.8% (n = 21) of the partial IN p32 gene, and 83.7% (n = 36) of the partial *env* gp41 showing positive bands in an agarose gel after electrophoresis. However, the PCR amplification of the partial *pol* PR/RT gene was considerably less successful at 34.8% (n = 15). PCR amplification of 10 samples were positive in all 4 gene regions and only one sample, 0116A, could not be amplified in any of the primers.

Thirty-seven (86.0%) of the *gag* p24, 13 (30.2%) of the *pol*, RT/PR, 18 (41.8%) of the *pol* IN and 35 (81.3%) of the *env* gp41 amplicons were successfully sequenced. The LANL QC tool indicated no stop codons, and no hypermutation was detected in any of the sequences.

### Preliminary subtype analysis using online tools

REGA and jpHMM online tools were used to assign subtypes to all the sequences and to detect possible recombinant forms. REGA 3.0 assigned all *gag, pol PR/RT, pol IN*, and *env* sequences to subtype C, except for *env* 0143A, which was assigned subtype B. Similar results were obtained with jpHMM, with the exception of the *IN* region of 0193A which was assigned as a CK recombinant form.

The SCUEAL subtyping of the *pol* PR/RT and IN gene fragments revealed that all the PR/RT and IN sequences were HIV-1 subtype C. Six of the sequences (18.75%) were intra-subtype C recombinant forms (Table 3).

**Table 3:** **Intra-subtype C recombinants detected using SQUEAL**

**Sample**	**Confidence**	**Recombination**	**Intra subtype recombination**	**Breakpoints**
0040_pol	0.749277	0.750944	0.750935	112 (111–113); 770 (769–771)
0042A_pol	0.917024	0.945140	0.945136	818 (797–839)
0143A_pol	0.691903	0.957455	0.957454	706 (701–711); 846 (844–848)
0173A_pol	0.736986	0.748732	0.748732	316 (250–382)
0040_IN	0.673024	0.690546	0.690544	551 (499–603)
0098A_IN	0.794292	0.999993	0.999127	172 (171–173); 383 (382–384); 736 (730–742)

### ML Phylogenetic inference

Model Selection (ML) using the BIC, implemented in MEGA, indicated the use of the (GTR + G + I) model for the *pol* and *env* regions and the use of the TN93 + G + I model for the *gag* region (Additional files 1, 2, 3 and 4: Table S1, Table S2, Table S3 and Table S4. Maximum Likelihood fits of 24 different nucleotide substitution models for gag, pol PR/RT, pol IN and env gp41, respectively). ML phylogenetic trees were inferred from the multiple sequence alignments, and branches with a bootstrap value of 70% or greater were considered reliable. None of the sub-genomic regions supported a monophyletic South African lineage.

**Figure 2:**
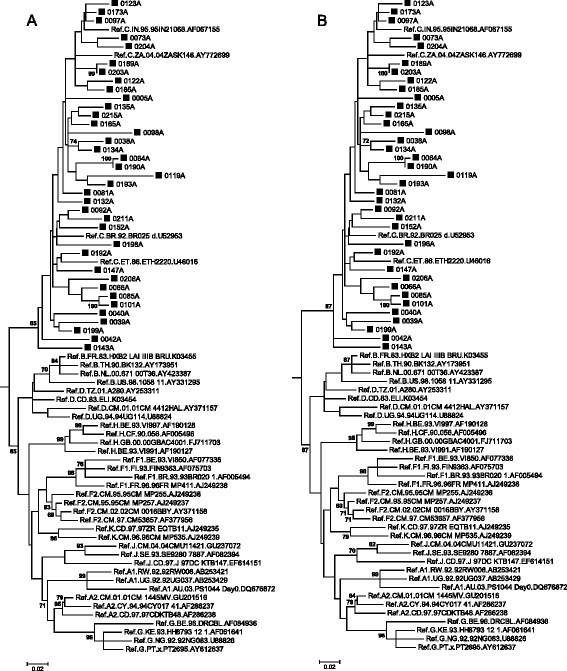
**Phylogenetic analysis of the partial** ***gag*** **gene, using MEGA 6. A**. The evolutionary history was inferred by using the ML method based on the GTR model. The tree with the highest log likelihood (−5337.9653) is shown. The percentage of trees in which the associated taxa clustered together is shown next to the branches. A discrete Gamma distribution was used to model evolutionary rate differences among sites (5 categories (+G, parameter = 1.2415)). The rate variation model allowed for some sites to be evolutionarily invariable ([+I], 52.1407% sites). The tree is drawn to scale, with branch lengths measured in the number of substitutions per site. The analysis involved 76 nucleotide sequences and included all codon positions. There were a total of 451 positions in the final dataset. **B**. The evolutionary history was inferred by using the ML method based on the Tamura-Nei model (TN93 + G + I). The tree with the highest log likelihood (−5340.4505) is shown. The percentage of trees in which the associated taxa clustered together is shown next to the branches. A discrete Gamma distribution was used to model evolutionary rate differences among sites (5 categories (+G, parameter = 1.1914)). The rate variation model allowed for some sites to be evolutionarily invariable ([+I], 51.4597% sites).

In the *gag* ML tree (Figure 2A and B) all the sequences clustered within subtype C. Except for slight differences in the bootstrap values, there were no differences in the *gag* tree topologies inferred with either the GTR + G + I or TN93 + G + I models. Interestingly the 2 outliers to the main subtype C cluster, 0042A and 0143A, were possible intra subtype C recombinants in the *pol* region. Sequence 0119A had a long branch and 3 sets of sequences, 0189A/0203A, 0064A/190A and 0085A/0101A clustered closely together. This may indicate that these samples may be a possible PCR contamination or that they are epidemiologically linked.

**Figure 3:**
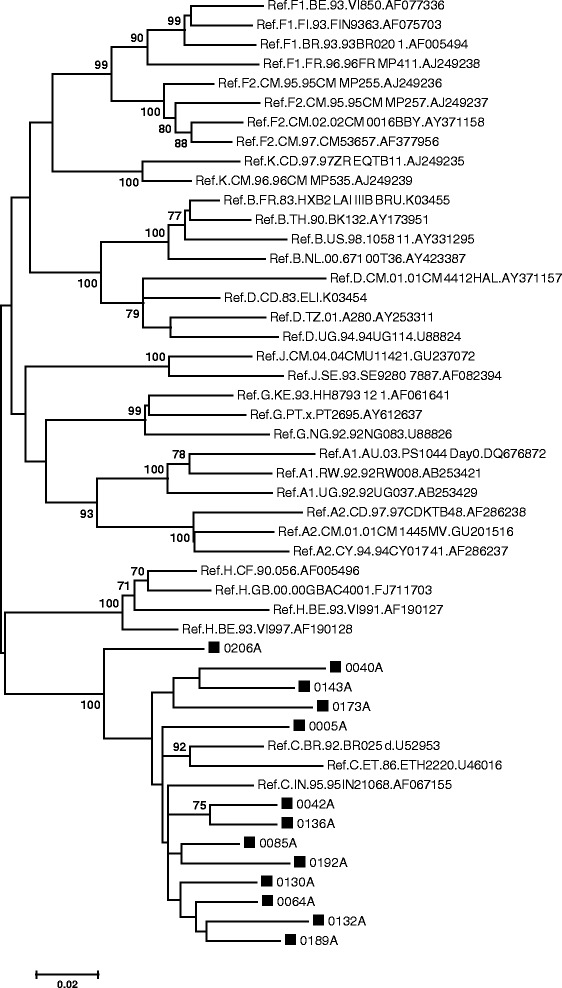
**Phylogenetic analysis of the partial** ***pol*** **gene, using MEGA 6.** The evolutionary history was inferred by using the ML method based on the GTR model. The tree with the highest log likelihood (−9574.7386) is shown. The percentage of trees in which the associated taxa clustered together is shown next to the branches. A discrete Gamma distribution was used to model evolutionary rate differences among sites (5 categories (+G, parameter = 1.1121)). The rate variation model allowed for some sites to be evolutionarily invariable ([+I], 45.2893% sites). The tree is drawn to scale, with branch lengths measured in the number of substitutions per site. The analysis involved 49 nucleotide sequences and included all codon positions. There were a total of 1062 positions in the final dataset.

**Figure 4:**
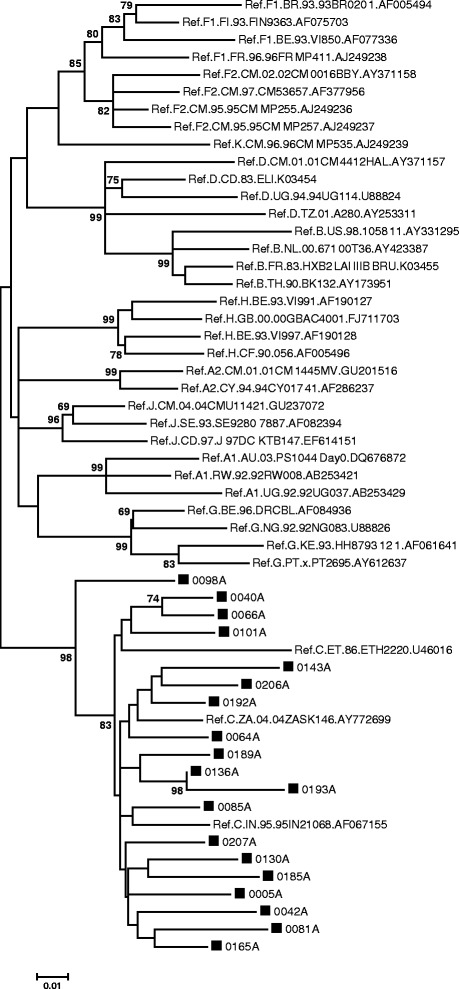
**Phylogenetic analysis of the** ***integrase*** **gene, using MEGA 6.** The evolutionary history was inferred by using the ML method based on the GTR model. The tree with the highest log likelihood (−7480.4899) is shown. The percentage of trees in which the associated taxa clustered together is shown next to the branches. A discrete Gamma distribution was used to model evolutionary rate differences among sites (5 categories (+G, parameter = 0.3186)). The rate variation model allowed for some sites to be evolutionarily invariable ([+I], 42.6830% sites). The tree is drawn to scale, with branch lengths measured in the number of substitutions per site. The analysis involved 55 nucleotide sequences and there were a total of 849 positions in the final dataset.

The ML phylogenetic tree for the *pol* PR/RT gene comprised 49 sequences and all the Mpumalanga sequences clustered with HIV-1 subtype C (Figure 3). The ML phylogenetic tree for the IN region contained 55 sequences and all Mpumalanga sequences clustered with HIV-1 subtype C reference sequences (Figure 4). Sequence 0098A clustered as an outlier to subtype C and SQUEAL indicated that the sequence is an intra-subtype C recombinant with 3 breakpoints. Sequence 0193A had a long branch and jpHMM indicated a possible CK recombinant form.

**Figure 5:**
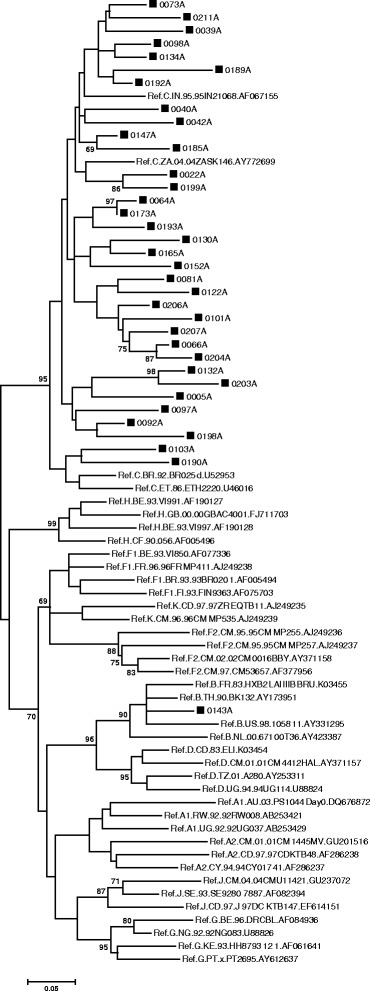
**Phylogenetic analysis of the partial** ***env*** **gene, using MEGA 6.** The evolutionary history was inferred by using the ML method based on the GTR model. The tree with the highest log likelihood (−7290.5638) is shown. The percentage of trees in which the associated taxa clustered together is shown next to the branches. A discrete Gamma distribution was used to model evolutionary rate differences among sites (5 categories (+G, parameter = 0.6134)). The rate variation model allowed for some sites to be evolutionarily invariable ([+I], 32.1349% sites). The tree is drawn to scale, with branch lengths measured in the number of substitutions per site. The analysis involved 74 nucleotide sequences and there were a total of 402 positions in the final dataset.

The ML phylogenetic tree for the *env* gp41 contained 74 sequences and all sequences, except for 0143A, clustered with HIV-1 subtype C sequences (Figure 5). Sequence 0143A clustered with subtype B in the *env* region and as an outlier to subtype C in the *gag* region. SQUEAL indicated that 0143A was an intra-subtype C recombinant in the *pol* region. This is the first indication of a putative unique BC recombinant sequence in Bushbuckridge, Mpumalanga.

### HIV-1 antiretroviral drug resistance mutations

Although the participants were from an antiretroviral treatment naïve cohort, some antiretroviral drug mutations were detected (Table 4). The NNRTI mutation K103N detected on the 0143A sequence causes high-level resistance to nevirapine (NVP), and efavirenz (EFV). The NNRTI mutation, E138A, detected on the 0143A sequence is a polymorphism that may contribute to reduced etravirine (ETR) and rilpivirine (RPV) susceptibility in combination with other NNRTI-resistance mutations. The K101E mutation found on the 0189A sequence causes intermediate resistance to NVP and low-level resistance to EFV, ETR, and RPV. No major PI mutations were detected in the Bushbuckridge, Mpumalanga sequences. The T74S minor PI mutation occurs in 5% of untreated persons with subtype C viruses and is associated with reduced NFV susceptibility [28-31].

**Table 4:** **HIV-1 drug resistance mutations detected in the Mpumalanga cohort**

**Isolate**	**Minor PI Mutations**	**NNRTI mutations**	**Integrase mutations**
0005A	None	None	L74I
0042A	T74S	None	None
0081A	None	None	E157Q
0143A	None	K103N	None
		E138A	
0173A	T74S	None	None
0189A	None	K101E	None
0192A	T74S	None	None
0198A	None	None	L74I
0206A	T74S	None	None

E157Q is an integrase polymorphic accessory mutation that is weakly selected in patients receiving raltegravir (RAL) and causes low level resistance to RAL and elvitegravir (EVG). L74I is an accessory mutation for integrase.

## Discussion

The investigation of the HIV subtype diversity of samples obtained from a cohort in Bushbuckridge, Mpumalanga revealed, first, that the HIV-1 from these samples belong almost entirely to HIV-1 subtype C with one BC recombinant; second, that the way in which the sequences derived from these samples cluster in phylogenetic trees suggests there has been multiple introductions of HIV-1 into Bushbuckridge; and third, the prevalence of antiretroviral drug resistance mutations and drug resistance-associated polymorphisms in Bushbuckridge is extremely low.

### Bushbuckridge HIV epidemic is predominantly subtype C, with one BC URF

The fact that the HIV samples from Bushbuckridge, Mpumalanga, belong almost entirely to subtype C is consistent not only with the explosive HIV-1 epidemic in southern Africa, but also it’s very limited subtype diversity. HIV-1 subtype C is the most common subtype, accounting for the majority of HIV infections in southern Africa [4,5], while subtype B is responsible for infections in MSM [9,12]. One putative subtype BC unique recombinant form was detected. This indicates that BC URFs are not only found in the Western Cape Province [15], but also in Mpumalanga province.

### Multiple introductions of HIV-1 into Bushbuckridge

The fact that HIV-1 subtype C sequences from South Africa tend to intermingle with HIV-1 subtype C sequences from Botswana, Malawi and Zambia suggests they may have a common evolutionary origin [32,33]. The possibility of an underlying common evolutionary origin of isolates in southern Africa is consistent with the history of the population dynamics of the southern African region. While the HIV-1 subtype C isolates from Brazil and Ethiopia tend to cluster separately, the fact that the subtype C isolate from India tends to cluster with the subtype C isolates from southern Africa [34,35] can be explained by the historical connections between the Indian subcontinent and southern Africa, which arises from the roles of both regions as former British colonial territories.

Countries in southern Africa in which adult national HIV prevalence rates exceeded 15% in 2007 were all linked by the migrant labor system. This system, which under pinned the population dynamics of both South Africa and the broader southern African region, was critical in shaping the patterns of population mobility and integration that characterizing the entire region. The migrant labor system was integral to the development and structure of the South African economy and apartheid. Botswana, Lesotho, Namibia, South Africa, Swaziland, Zambia, and Zimbabwe, were all historically linked through the migrant labor system that brought men from as far as Zambia and Malawi to the mines initially on the Reef and subsequently elsewhere in the country [36-39]. Migrants are more vulnerable to HIV infection than people who hardly move, both in southern Africa as in other African countries [40-42]. A 1985 survey of workers in the gold mines originating from the entire southern African region found HIV prevalence to be very low among South African miners but among Malawian miners prevalence was already at 3% [36]. High infection levels are being found in Gaza province in Mozambique, where large numbers of migrants working in South Africa originate [43]. Before and after independence foreign migrant workers also crossed borders to work in mines in Namibia, Botswana, Zambia, and Zimbabwe [44-46].

Many of the countries in southern African with explosive HIV/AIDS epidemic are also landlocked, which entails that the region’s road transport networks does not only link these landlocked countries to the ports in Durban, Richards Bay and Maputo, but also facilitate the rapid spread of HIV in the region by ensuring the sexual networks that drive the epidemic transcend national boundaries. The Ehlanzeni District in Mpumalanga Province straddles the Maputo Corridor, a major trade route which connects the Gauteng, Limpopo, and Mpumalanga provinces of South Africa with Maputo, the capital of Mozambique that also has a major port. In Mozambique, HIV is spreading more rapidly in provinces linked by major transport routes to Malawi, South Africa and Zimbabwe. High infection rates have been found in Sofia province, which is traversed by Zimbabwe’s main export route [43].

The peculiarly explosive HIV-1 epidemic in southern Africa could also stem from the unique biological properties of subtype C. HIV-1 subtype C has an additional NF-binding site in the long terminal repeat (LTR), a prematurely truncated Rev protein, a 5′-amino-acid insertion in Vpu, and a more active, catalytically efficient protease, which may influence viral gene expression and alter the transmissibility and pathogenesis of subtype C isolates [31,47-52]. These unique biological properties, including those related to viral entry and pathogenesis such as the CCR5 and non-syncytium-inducing phenotype, may account for the explosive epidemic of HIV-1 subtype C in southern Africa [53-55]. However, the additional NF-κB site in HIV-1 subtype C may be biologically inactive, and enhanced activity of these individual functions may still not be sufficient to overcome the decreased replicative capacity of the CCR5-tropic non-syncytium-inducing phenotype [53].

### Drug resistance mutations and polymorphisms

Combination antiretroviral therapy can suppress HIV-1 replication to undetectable levels with concomitant significant clinical outcomes. However, suboptimal suppression HIV-1 replication can result in the emergence of drug resistant virus strains. HIV-1 isolates that have acquired mutations conferring reduced susceptibility to antiretroviral drugs can be can be transmitted, potentially limiting options for first line therapy in untreated individuals [56]. The proportion of patients without prior antiretroviral therapy and who are infected with a virus resistant to at least one antiretroviral drug in Australia, Europe, Japan and the United States of America is 10% to 17%, while data between 2006 and 2010 suggests that transmitted antiretroviral drug resistance among those starting antiretroviral treatment in low- and middle-income countries increasing [2].

South Africa has the largest antiretroviral treatment program in the world. Besides its unprecedented scale, the antiretroviral treatment programme in South Africa is also being rolled out rapidly, such that while only 833653 adults and 86270 children were on antiretroviral treatment through the public sector in South Africa by the end of 2009, the number of those on treatment by 2012 had increased to 2010340 adults and 140541 children [2,3,16].

While the HIV-1 sequences used in this study are derived from treatment-naïve participants from Bushbuckridge, Mpumalanga, the K103N antiretroviral drug resistance mutation was detected. This suggests the participants from Bushbuckridge, Mpumalanga may either have undergone antiretroviral treatment or that they were infected with antiretroviral drug resistant strains [28-31]. The E138A mutation selected for by riplivirine/etravirine, must also be a transmitted mutation. Both riplivirine/etravirine are not part of the first and second line ART regimens in South Africa, while etravirine is part of the third line regimen. Patients in Mpumalanga only started receiving third line ART 2013.

### Limitations of this study

The limitations of the study include a relatively small sample size; DNA amplification was not successful for up to 71% of the samples of the partial *pol* PR/RT sub-genomic region; use of partial gene regions to assign viral subtypes, potentially allowing recombinant viruses to be missed, the use of direct; population sequencing may result in the lack of detection of minority-population viruses; which can lead to an underestimation of viral diversity and drug resistance mutations.

## Conclusions

HIV diversity may have implications for diagnosis, pathogenesis, transmission, clinical management and vaccine development. Phylogenetic analysis of HIV sequence diversity has allowed vital insights into the origin, evolution and spread of HIV, which suggests it is imperative to maintain HIV-1 molecular epidemiology surveillance. The extensive population mobility arising from the historical and structural migrant labor system characterizing South Africa, and the concomitant overlapping of sexual networks, seems to have precluded the possibility of distinct geographical lineages developing. However, the demise of apartheid, in particular the end of influx control measures, may have a significant impact on patterns of population mobility and settlement in South Africa, which in turn may affect the patterns of transmission of HIV and ultimately it’s evolution. The possible emergence of various HIV-1 recombinants could suggest that the migration of people into South Africa from Central, West and eastern Africa could also impact on the character and dynamics of the HIV/AIDS epidemic in South Africa.

## Additional files

Additional file 1: Table S1. ML fit of 24 different nucleotide substitution models for HIV-1 *gag* dataset. Additional file 2: Table S2. ML fit of 24 different substitution models for HIV-1 *pol* PR/RT dataset. Additional file 3: Table S3. ML fit of 24 different substitution models for HIV-1 *pol* IN dataset. Additional file 4: Table S4. ML fit of 24 different substitution models for HIV-1 *env* gp41 dataset.

## References

[1] Barré-Sinoussi F, Chermann JC, Rey F, Nugeyre MT, Chamaret S, Gruest J, Chamalet S, Gruest J, Dauguet C, Axler-Blin C (1983). Isolation of a T-lymphotropic retrovirus from a patient at risk for acquired immune deficiency syndrome (AIDS). Science.

[2] UNAIDS: The gap report. Geneva 2014. http://www.unaids.org/en/resources/documents/2014/name,97466,en.asp

[3] Shisana O, Rehle T, Simbayi LC, Zuma K, Jooste S, Zungu N, Labadarios D, Onoya D, Van Zyl J, Wabiri N (2014). South African National HIV prevalence, incidence and behaviour survey, 2012.

[4] Van Harmelen JH, Van der Ryst E, Loubser AS, York D, Madurai S (1999). A predominantly HIV-1 subtype C-restricted epidemic in South African urban populations. AIDS Res Hum Retroviruses.

[5] Williamson C, Engelbrecht S, Lambrick M, Van Rensburg EJ, Wood R, Bredell W, Williamson A-L (1995). HIV-1 subtypes in different risk groups in South Africa. Lancet.

[6] Engelbrecht S, Laten JD, Smith TL, van Rensburg EJ (1995). Identification of *env* subtypes in fourteen HIV type 1 isolates from South Africa. AIDS Res Hum Retroviruses.

[7] Loxton AG, Treurnicht F, Laten A, Van Rensburg EJ, Engelbrecht S (2005). Sequence analysis of near full-length HIV type 1 subtype D primary strains isolated in Cape Town, South Africa, from 1984 to 1986. AIDS Res Hum Retroviruses.

[8] Jacobs GB, De Beer C, Fincham JE, Adams V, Dhansay MA, van Rensburg EJ, Engelbrecht S (2006). Serotyping and genotyping of HIV-1 infection in residents of Khayelitsha, Cape Town, South Africa. J Med Virol.

[9] Jacobs GB, Loxton AG, Laten A, Robson B, van Rensburg EJ, Engelbrecht S (2009). Emergence and diversity of different HIV-1 subtypes in South Africa, 2000–2001. J Med Virol.

[10] Bredell H, Hunt G, Casteling A, Cilliers T, Rademeyer C, Coetzer M, Miller S, Johnson D, Tiemessen CT, Martin DJ, Williamson C, Morris L (2002). HIV-1 subtype A, D, G, AG and unclassified sequences identified in South Africa. AIDS Res Hum Retroviruses.

[11] Iweriebor BC, Bessong PO, Mavhandu LG, Masebe TM, Nwobegahay J, Moyo SR, Mphahlele JM (2011). Genetic analysis of the near full-length genome of an HIV type 1 A1/C unique recombinant form from northern South Africa. AIDS Res Hum Retroviruses.

[12] Middelkoop K, Rademeyer C, Brown BB, Cashmore TJ, Marais JC, Scheibe AP, Bandawe GP, Myer L, Fuchs JD, Williamson C, Bekker LG (2014). Epidemiology of HIV-1 subtypes among men who have sex with men in Cape Town, South Africa. J Acquir Immune Defic Syndr.

[13] Papathanasopoulos MA, Cilliers T, Morris L, Mokili JL, Dowling W, Birx DL, McCutchan FE (2002). Full-length genome analysis of HIV-1 subtype C utilizing CXCR4 and intersubtype recombinants isolated in South Africa. AIDS Res Hum Retroviruses.

[14] Wilkinson E, Engelbrecht S (2009). Molecular characterization of non-subtype C and recombinant HIV-1 viruses from Cape Town, South Africa. Infect Genet Evol.

[15] Jacobs GB, Wilkinson E, Isaacs S, Spies G, de Oliveira T, Seedat S, Engelbrecht S (2014). HIV-1 subtypes B and C unique recombinant forms (URFs) and transmitted drug resistance identified in the Western Cape Province, South Africa. PLoS One.

[16] South African Government (2013). The 2012 National Antenatal Sentinel HIV and Herpes Simplex Type-2 Prevalence Survey in South Africa.

[17] South African Government (2005). Bushbuckridge Nodal Economic Development Profile Mpumalanga South Africa.

[18] Swanson P, Devare SG, Hackett J (2003). Molecular characterization of 39 HIV-1 isolates representing group M (subtypes A-G) and group O: Sequence analysis of gag p24, pol integrase, and env gp41. AIDS Res Hum Retroviruses.

[19] Jacobs GB, Laten A, van Rensburg EJ, Bodem J, Weissbrich B, Rethwilm A, Preiser W, Engelbrecht S (2008). Phylogenetic diversity and low level antiretroviral resistance mutations in HIV type 1 treatment-naive patients from Cape Town, South Africa. AIDS Res Hum Retroviruses.

[20] Plantier JC, Dachraoui R, Lemee V, Gueudin M, Borsa-Lebas F, Caron F, Simon F (2005). HIV-1 resistance genotyping on dried serum spots. AIDS.

[21] Pineda-Peña AC, Faria NR, Imbrechts S, Libin P, Abecasis AB, Deforche K, Gómez-López A, Camacho RJ, de Oliveira T, Vandamme AM (2013). Automated subtyping of HIV-1 genetic sequences for clinical and surveillance purposes: performance evaluation of the new REGA version 3 and seven other tools. Infect Genet Evol.

[22] Schultz AK, Zhang M, Bulla I, Leitner T, Korber B, Morgenstern B, Stanke M (2009). jpHMM: Improving the reliability of recombination prediction in HIV-1. Nucleic Acids Res.

[23] Kosakovsky Pond SL, Posada D, Stawiski E, Chappey C, Poon AF, Hughes G, Fearnhill E, Gravenor MB, Leigh Brown AJ, Frost SD (2009). An evolutionary model-based algorithm for accurate phylogenetic breakpoint mapping and subtype prediction in HIV-1. PLoS Comput Biol.

[24] Katoh K, Standley DM (2013). MAFFT multiple sequence alignment software version 7: improvements in performance and usability. Mol Biol Evol.

[25] Tamura K, Stecher G, Peterson D, Filipski A, Kumar S (2013). MEGA6: Molecular Evolutionary Genetics Analysis version 6.0. Mol Biol Evol.

[26] Felsenstein J (1985). Confidence limits on phylogenies: An approach using the bootstrap. Evolution.

[27] Rhee SY, Gonzales MJ, Kantor R, Betts BJ, Ravela J, Shafer RW (2003). Human immunodeficiency virus reverse transcriptase and protease sequence database. Nucleic Acids Res.

[28] Barth RE, Wensing AM, Tempelman HA, Moraba RR, Schuurman R (2008). Rapid accumulation of non-nucleoside reverse transcriptase inhibitor-associated resistance: evidence of transmitted resistance in rural South Africa. AIDS.

[29] Cane PA, De Ruiter A, Rice P, Wiselka M, Fox R, Pillay D (2001). Resistance associated mutations in the human immunodeficiency virus type 1 subtype C protease gene from treated and untreated patients in the United Kingdom. J Clin Microbiol.

[30] Soares EA, Santos AF, Gonzalez LM, Lalonde MS, Denis M, Tebit DM, Tanuri A, Arts EJ, Soares M (2009). Mutation T74S in HIV-1 subtype B and C proteases resensitizes them to ritonavir and indinavir and confers fitness advantage. J Antimicrob Chemother.

[31] Velazquez-Campoy A, Todd MJ, Vega S, Freire E (2001). Catalytic efficiency and vitality of HIV-1 proteases from African viral subtypes. Proc Natl Acad Sci U S A.

[32] Novitsky VA, Montano MA, McLane MF, Renjifo B, Vannberg F, Foley BT, Ndung’u TP, Rahman M, Makhema MJ, Marlink R, Essex M (1999). Molecular cloning and phylogenetic analysis of human immunodeficiency virus type 1 subtype C: a set of 23 full-length clones from Botswana. J Virol.

[33] Rousseau CM, Birditt BA, McKay AR, Stoddard JN, Lee TC, McLaughlin S, Moore SW, Shindo N, Learn GH, Korber BT, Brander C, Goulder PJ, Kiepiela P, Walker BD, Mullins JI (2006). Large-scale amplification, cloning and sequencing of near full-length HIV-1 subtype C genomes. J Virol Methods.

[34] Gaschen B, Taylor J, Yusim K, Foley B, Gao F, Lang D, Novitsky V, Haynes B, Hahn BH, Bhattacharya T, Korber B (2002). Diversity considerations in HIV-1 vaccine selection. Science.

[35] Shankarappa R, Chatterjee R, Learn GH, Neogi D, Ding M, Roy P, Ghosh A, Kingsley L, Harrison L, Mullins JI, Gupta P (2001). Human immunodeficiency virus type 1 env sequences from Calcutta in eastern India: identification of features that distinguish subtype C sequences in India from other subtype C sequences. J Virol.

[36] Abdool Karim Q, Abdool Karim SS (2002). The evolving HIV epidemic in South Africa. Int J Epidemiol.

[37] Bauer G, Taylor SD (2005). Politics in Southern Africa: state and society in transition.

[38] Dusheiko GM, Brink BA, Conradie JD, Marimuthu T, Sher R (1989). Regional prevalence of hepatitis B, delta, and human immunodeficiency virus infection in southern Africa: a large population survey. Am J Epidemiol.

[39] Huang KH, Goedhals D, Fryer H, van Vuuren C, Katzourakis A, De Oliveira T, Brown H, Cassol S, Seebregts C, McLean A, Klenerman P, Phillips R, Frater J, Bloemfontein-Oxford Collaborative Group (2009). Prevalence of HIV type-1 drug-associated mutations in pre-therapy patients in the Free State South Africa. Antivir Ther.

[40] Abdool Karim SS, Abdool Karim Q (1992). Changes in HIV seroprevalence in a rural black community in KwaZulu Natal. S Afr Med J.

[41] Decosas J, Kane F, Anarfi JK, Sodji KD, Wagner HU (1995). Migration and AIDS. Lancet.

[42] Lurie MN, Williams BG, Zuma K, Mkaya-Mwamburi D, Garnett G, Sturm AW, Sweat MD, Gittelsohn J, Abdool Karim SS (2003). The impact of migration on HIV-1 transmission in South Africa: a study of migrant and nonmigrant men and their partners. Sex Transm Dis.

[43] Ministry of Health, Mozambique: Ministry of Health National Control Program ITS/HIV-SIDA (2005). Relatório sobre a Revisão dos Dadosde Vigilância Epidemiológica do HIV-Ronda 2004 [Report regarding the revision of the epidemiological surveillance data on HIV - Round 2004].

[44] Lurie M (2000). Migration and AIDS in southern Africa: a review. S Afr J Sci.

[45] Lurie MN, Williams BG, Zuma K, Mkaya-Mwamburi D, Garnett GP, Sweat MD, Gittelsohn J, Abdool Karim SS (2003). Who infects whom? HIV-1 concordance and discordance among migrant and non-migrant couples in South Africa. AIDS.

[46] Ramjee G, Gouws E (2002). Prevalence of HIV among truck drivers visiting sex workers in KwaZulu-Natal, South Africa. Sex Transm Dis.

[47] De Oliveira T, Engelbrecht S, Van Rensburg E, Gordon M, Bishop K, Zur Megede J, Barnett SW, Cassol S (2003). Variability at HIV- 1 subtype C protease cleavage sites and indication of viral fitness?. J Virol.

[48] Gao F, Robertson DL, Carruthers CD, Morrison SG, Jian B, Chen Y, Barre-Sinoussi F, Girard M, Srinivasan A, Abimiku AG, Shaw GM, Sharp PM, Hahn BH (1998). A comprehensive panel of near-full-length clones and reference sequences for non-subtype B isolates of human immunodeficiency virus type1. J Virol.

[49] Hunt G, Tiemessen CT (2000). Occurrence of additional NF-κB-binding motifs in the long terminal repeat region of South African HIV type 1 subtype C Isolates. AIDS Res Human Retroviruses.

[50] McCormick-Davis C, Dalton SB, Singh DK, Stephens EB (2000). Comparisons of Vpu sequences from diverse geographical isolates of HIV type 1 identifies the presence of highly variable domains additional invariant amino acids and a signature sequence motif common to subtype C isolates. AIDS Res Hum Retroviruses.

[51] Rodenburg CM, Li Y, Trask SA (2001). Near full-length clones and reference sequences for subtype C isolates of HIV type 1 from three different continents. AIDS Res Hum Retroviruses.

[52] Ball SC, Abraha A, Collins KR, Marozsan AJ, Baird H, Quiñones-Mateu ME, Penn-Nicholson A, Murray M, Richard N, Lobritz M, Zimmerman PA, Kawamura T, Blauvelt A, Arts EJ (2003). Comparing the *ex vivo* fitness of CCR5- tropic human immunodeficiency virus type 1 isolates of subtypes B and C. J Virol.

[53] Peeters M, Vincent R, Perret JL, Lasky M, Patrel D, Liegeois F, Courgnaud V, Seng R, Matton T, Molinier S, Delaporte E (1999). Evidence for differences in MT2 cell tropism according to genetic subtypes of HIV-1: syncytium-inducing variants seem rare among subtype C HIV-1 viruses. J Acquir Immune Defic Syndr Hum Retrovirol.

[54] Ping LH, Nelson JA, Hoffman IF, Schock J, Lamers SL, Goodman M, Vernazza P, Kazembe P, Maida M, Zimba D, Goodenow MM, Eron JJ, Fiscus SA, Cohen MS, Swanstrom R (1999). Characterization of V3 sequence heterogeneity in subtype C human immunodeficiency virus type 1 isolates from Malawi: underrepresentation of X4 variants. J Virol.

[55] Gifford RJ, Liu TF, Rhee SY, Kiuchi M, Hue S, Pillay D, Shafer RW (2009). The calibrated population resistance tool: standardized genotypic estimation of transmitted HIV-1 drug resistance. Bioinformatics.

[56] Shafer RW, Rhee SY, Bennett DE (2008). Consensus drug resistance mutations for epidemiological surveillance: basic principles and potential controversies. Antivir Ther.

